# Updated Adjuvant Chemotherapy for Gastric Cancer

**DOI:** 10.3390/jcm12216727

**Published:** 2023-10-24

**Authors:** Toshizo Takayama, Yasushi Tsuji

**Affiliations:** 1 Department of Medical Oncology, Tonan Hospital, Sapporo 060-0004, Japan; 2 Department of Medical Oncology, Daido Hospital, Nagoya 457-8511, Japan

**Keywords:** adjuvant chemotherapy, perioperative treatment, gastric cancer

## Abstract

Surgical resection is currently the best curative approach for gastric cancer (GC); however, the prognosis of patients with advanced GC remains poor even with curative resection. For this reason, perioperative chemotherapy has been combined with surgery to reduce the risk of postoperative recurrence. Standard perioperative chemotherapy for resectable advanced GC varies from region to region. Postoperative S-1 therapy was standardized via the ACTS-GC study in East Asia, perioperative ECF (Epirubicin + Cisplatin + Fluorouracil) was standardized via the MAGIC study in Europe, and postoperative chemoradiotherapy was standardized via the US intergroup study in North America. Since then, more intensive regimens have been developed. In recent years, perioperative therapy using novel agents, such as molecular-targeted drugs and immune checkpoint inhibitors (ICIs), has also been tested and evaluated in the three major regions (East Asia, Europe, and North America) with promising results. Perioperative chemotherapy has become an integral part of many treatment strategies and requires continued research and evaluation.

## 1. Introduction

GC is a highly malignant cancer with high morbidity and mortality rates worldwide. With more than 1 million newly diagnosed cases, GC remains the leading cause of cancer death every year [1].

Surgical resection is currently the most reliable curative approach for GC. Although improvements in surgical techniques and perioperative care have led to decreased surgical morbidity and mortality, the prognosis of patients with advanced GC remains poor even after complete resection [2,3]. The reason is that minimal residual disease (MRD) can remain in the body even after curative resection. Patients with MRD have a high probability of disease recurrence.

Recently, circulating tumor DNA (ctDNA) has been recognized as an important biomarker to assess the recurrence risk of bladder, breast, and colorectal cancers [4,5,6,7]. Postoperative ctDNA can be considered an MRD biomarker offering useful prognostic information regarding recurrence [8]. It has also been reported that ctDNA may be involved in the recurrence of resectable GC [9,10,11]. However, ctDNA assays have not been established in actual clinical practice and need to be further developed and streamlined.

Perioperative chemotherapy has been used worldwide since the 2000s to minimize the risk of recurrence by eliminating MRD.

In recent years, various clinical trials have been conducted in all regions to improve survival by enhancing perioperative chemotherapy.

This article summarizes the perioperative chemotherapy approaches by region.

## 2. East Asia

### 2.1. Postoperative Chemotherapy in East Asia (Table 1 and Table 2)

In East Asia, radical gastrectomy with D2 lymph node resection has been the standard surgery for GC. Many studies have investigated postoperative adjuvant chemotherapy, and it is now also considered a standard treatment. 

This section summarizes pivotal clinical trials on postoperative chemotherapy.

#### 2.1.1. The ACTS-GC Study

The first convincing evidence from a randomized clinical study for the efficacy of S-1 adjuvant chemotherapy was the phase III Adjuvant Chemotherapy Trial for Gastric Cancer (ACTS-GC) study [12]. 

In this study, 1059 patients with stage II-III GC who underwent curative gastrectomy with D2 lymph node dissection were enrolled. Patients were randomly assigned to either the S-1 chemotherapy group or the surgery-only group. The primary endpoint was overall survival (OS). The 3-year OS rate was 80.1% in the S-1 group and 70.1% in the surgery-only group. The death hazard ratio (HR) in the S-1 group, as compared with the surgery-only group, was 0.68 (95% confidence interval (CI): 0.52–0.87; *p* = 0.003). The HR for recurrence-free survival (RFS) at 3 years was 0.62 (95% CI: 0.50–0.77). This analysis was updated after 5 years of follow-up and demonstrated consistent results [13], showing a significant 10.6% improvement in 5-year OS with adjuvant S-1 chemotherapy group compared to the surgery-only group (71.7 vs. 61.1%, HR 0.669, 95% CI: 0.540–0.828), as well as a 12.3% improvement in 5-year RFS compared to the surgery-only group (65.4 vs. 53.1%, HR 0.653, 95% CI: 0.537–0.793). Along with the marked efficacy, low rates of grades 3 and 4 toxicities were observed (6.0% anorexia, 3.7% nausea, and 3.1% diarrhea). Based on this study, S-1 was established as a standard regime of postoperative adjuvant chemotherapy in East Asia for patients with locally advanced GC who underwent radical surgery.

However, it is not clear whether the efficacy of adjuvant S-1, as demonstrated in the ACTS-GC study, is also achievable in Western populations where this drug is poorly tolerated because of CYP2A6 polymorphisms, by which tegafur present in S-1 transforms in the body to 5-FU [14].

#### 2.1.2. The CLASSIC Study

Capecitabine is an oral fluoropyrimidine agent administered worldwide. The Capecitabine and Oxaliplatin Adjuvant Study in Stomach Cancer (CLASSIC) study was a phase III study that was conducted in East Asia. This study reported a significant efficacy regarding disease-free survival (DFS) after 6 months of adjuvant (postoperative) capecitabine plus oxaliplatin (XELOX) following gastrectomy with D2 lymph node dissection compared to surgery alone [15].

In this study, 1035 patients from 37 Asian centers with stages II–IIIB gastric adenocarcinoma who underwent curative gastrectomy with D2 lymph node dissection were randomly assigned to either the surgery-only group or the adjuvant XELOX group. Patients assigned to the XELOX group had significantly improved 3-year DFS (74 vs. 59%, HR 0.56, 95% CI: 0.44–0.72), with only a borderline statistically significant improvement in 3-year OS rate (83 vs. 78%, HR 0.72, 95% CI: 0.52–1.00) at a median follow-up of 34.2 months.

However, with longer follow-up, the improved OS with XELOX became statistically significant (5-year OS 78 vs. 69%, HR 0.66, 95% CI: 0.51–0.85) [16]. The adjuvant XELOX group had a higher rate of grades 3 and 4 toxicities (56 vs. 6% for the surgery-only group), most commonly neutropenia, nausea, vomiting, thrombocytopenia, and anorexia, requiring chemotherapy dose modifications in 90% of patients. Only 67% of patients assigned to the adjuvant XELOX group completed 6 months of chemotherapy compared to 78% in the ACTS-GC study. The CLASSIC study established XELOX as one of the new treatment options for postoperative chemotherapy in GC patients who underwent gastrectomy with D2 lymph node dissection.

#### 2.1.3. The JACCRO GC-07 Study (START-2)

Docetaxel (DTX) has shown efficacy in advanced or recurrent GC not only as a monotherapy but also in combination with fluoropyrimidine plus cisplatin [17,18,19,20,21,22]. 

The JACCRO GC-07 was a phase III study conducted to prove the superiority of postoperative S-1 plus DTX. This study enrolled patients who underwent curative resection via D2 or more extensive gastrectomy and were diagnosed with stage III GC. They were randomly assigned to either postoperative S-1 plus DTX or postoperative S-1 alone [23].

Chemotherapy was to be started within 6 postoperative weeks. Patients assigned to the S-1 plus docetaxel group were treated with S-1 on days 1 to 14 of a 3-week cycle during the first course. In the second to seventh courses, patients received intravenous infusion of DTX on day 1 of each cycle and S-1 on days 1 to 14 of a 3-week cycle. From the eighth course, treatment with S-1 continued on days 1 to 28 of 6-week cycles for up to 1 year. Patients assigned to the S-1 group were treated with S-1 every 6-week cycle for up to 1 year. The primary endpoint was 3-year RFS.

The interim analysis of RFS proved the superiority of the S-1 plus DTX (HR 0.632; 99.99% CI: 0.400–0.998; *p* < 0.001), with a 3-year RFS of 66% (95% CI: 59–73%) in the S-1 plus DTX group and 50% (95% CI: 41–58%) in the S-1 group. Median RFS was not reached in the S-1 plus DTX group, whereas in the S-1 group, it was 34.5 months (95% CI: 29.5 months—not reached).

The S-1 plus DTX group had a higher incidence of grades 3 or 4 toxicities, especially neutropenia and leukopenia, than the S-1 group, but all toxicities were manageable.

#### 2.1.4. The ARTIST 2 Study

In North America, the Intergroup-0116 study [24] reported the effectiveness of adjuvant chemoradiotherapy. However, the effectiveness of chemoradiotherapy in patients who underwent gastrectomy with D2 lymph node dissection remains controversial, despite positive data being reported [25]. This study compared three adjuvant regimens: oral S-1 for 1 year, S-1 plus oxaliplatin (SOX) for 6 months, and SOX plus radiotherapy 45 Gy (SOXRT) in a 1:1:1 ratio [26]. The primary endpoint was 3-year DFS; a 33% reduction in the HR for DFS with SOX or SOXRT, when compared with S-1, was considered clinically meaningful. 

In this study, 546 patients were randomly assigned to either the S-1, SOX, or SOXRT group. After a median follow-up of 47 months, the 3-year DFS rates were 64.8, 74.3, and 72.8% in the S-1, SOX, and SOXRT groups, respectively. HR for DFS in the control arm (S-1 group) was lower than that in the SOX and SOXRT groups: S-1 versus SOX, 0.692 (*p* = 0.042); S-1 versus SOXRT, 0.724 (*p* = 0.074). There was no statistical difference in DFS between SOX and SOXRT (HR 0.971; *p* = 0.879). The regimens were generally well tolerated, and adverse events were manageable.

This study showed the efficacy of SOX or SOXRT in patients who underwent curative gastrectomy with D2 lymph node dissection, specifically those with pStage II or III node-positive GC. However, this study did not confirm the efficacy of radiotherapy.

#### 2.1.5. Final Notes

Postoperative adjuvant chemotherapy can improve the cure rate after resection and is, thus, recommended for pStage II to III. Although combination therapy is recommended for pStage III, it is not clear what therapy is more effective because there is no direct comparison of the three regimens (S-1/docetaxel, SOX, and XELOX).

**Table 1:** Pivotal randomized controlled clinical trial for postoperative chemotherapy in East Asia.

Study	N	Published Year	Area	Setting	Disease Type	Recommended Resection	Treatment	Survival	HR	Reference
ACTS-GC	530	2007	Japan	Stage II/III	gastric cancer 100%	D2	Surgery alone	5-year OS: 61%	0.669	[12,13]
	529				AC 100%		S-1	5-year OS: 72%		
CLASSIC	515	2014	Korea	Stage II/IIIB	gastric cancer 98%,GEJ 2%	D2	Surgery	5-year OS: 69%	0.66	[15,16]
	520				AC 100%		XELOX	5-year OS: 78%		
JACCRO GC-07	459	2019	Japan	Stage III	gastric cancer 100%	D2	S-1	3-year RFS:50%	0.632	[23]
	454				AC 100%		S-1 + DTX	3-year RFS:66%		
ARTIST 2	182	2021	Korea	Stage II/III	gastric cancer 100%	D2	S-1	3-year DFS:65%	-	[26]
	181						SOX	3-year DFS:74%	0.692	
					AC 100%					
	183						SOX + RT (45 Gy)	3-year DFS:73%	0.724	

GEJ—gastroesophageal junction; AC—adenocarcinoma; OS—overall survival; RFS—recurrence-free survival; DFS—disease-free survival; XELOX—capecitabine and oxaliplatin; SOX—S-1 and oxaliplatin; RT—radiotherapy; HR—hazard ratio.

**Table 2:** Postoperative chemotherapy regimens in East Asia.

Regimen	Frequency	Drug	Dose		Period
**S-1**	6 weeks	S-1	40–80 mg/body, twice daily	days 1–28	every 6 weeks for 1 year
**XELOX (CapeOx)**	3 weeks	Capecitabine	1000 mg/m^2^, twice daily	days 1–14	8 cycles
		Oxaliplatine	130 mg/m^2^	day 1	
**S-1 + DTX**	(1) 3 weeks	S-1	40–80 mg/body, twice daily	days 1–14	1 cycle
	(2) 3 weeks	S-1	40–80 mg/body, twice daily	days 1–14	6 cycles
		Docetaxel	40 mg/m^2^	day 1	
	(3) 6 weeks	S-1	40–80 mg/body, twice daily	days 1–28	every 6 weeks for total 1 year
**SOX**	3 weeks	S-1	40–80 mg/body, twice daily	days 1–14	8 cycles
		Oxaliplatine	130 mg/m^2^	day 1	
**SOX + RT**	(1) 3 weeks	S-1	40–80 mg/body, twice daily	days 1–14	2 cycles
		Oxaliplatine	130 mg/m^2^	day 1	
	(2) 5 weeks	S-1	40–80 mg/body, twice daily	5 days a week, over 5 weeks	
		Radiotherapy	45 Gy/25 Fr		
	(3) weeks	S-1	40–80 mg/body, twice daily	days 1–14	4 cycles
		Oxaliplatine	130 mg/m^2^	day 1	

### 2.2. Neoadjuvant Chemotherapy in East Asia (Table 3 and Table 4)

As mentioned above, postoperative chemotherapy is the standard treatment in East Asia.

On the other hand, neoadjuvant chemotherapy (NAC) has also been investigated, and the results are reported below.

#### 2.2.1. JCOG0501

Even with standard perioperative therapy and curative surgery, GCs with linitis plastica (Borrmann type 4) and large ulcero-invasive-type (≥8 cm, Borrmann type 3) have a very poor prognosis. JCOG0501 is a randomized phase III study conducted to evaluate the survival benefit of neoadjuvant (preoperative) chemotherapy of SP (S-1 + CDDP) for postoperative S-1 in resectable type 4 and large type 3 GCs in Japan [27].

Patients assigned to Arm A underwent total or distal gastrectomy with D2 or D3 lymph node dissection. Patients in Arm B received preoperative chemotherapy with SP (S-l administered for the first 3 weeks of a 4-week course, and Cisplatin administered on day 8 of each course). All patients who underwent curative gastrectomy received postoperative chemotherapy with S-1 orally on days 1–28 of each 6-week cycle for 1 year.

A total of 300 patients were enrolled in this study. The 3-year OS rates were 62.4% (95% CI: 54.1–69.6) in Arm A and 60.9% (95% CI: 52.7–68.2) in Arm B. The HR in Arm B, as compared with Arm A, was 0.916 (95% CI: 0.679–1.236). 

For Borrmann type 4 or large type 3 GC, the addition of preoperative chemotherapy with SP did not show a survival benefit. The *Japanese Gastric Cancer Treatment Guidelines* do not recommend neoadjuvant chemotherapy, and still continue to recommend D2 gastrectomy followed by postoperative chemotherapy [28].

#### 2.2.2. PRODIGY

This study was conducted to evaluate whether neoadjuvant (preoperative) docetaxel, oxaliplatin, and S-1 (DOS) followed by surgery and adjuvant (postoperative) S-1 could improve outcomes versus standard treatment (surgery and adjuvant S-1) in patients with resectable GC in Korea [29].

Patients with histologically confirmed primary gastric or gastroesophageal junction adenocarcinoma (clinical TNM staging: T2-3N1 or T4N any) were enrolled. They were randomly assigned to either D2 surgery followed by adjuvant S-1 (SC group) or neoadjuvant DOS before D2 gastrectomy, followed by adjuvant S-1 (CSC group). The primary endpoint was PFS.

In this study, 266 patients were assigned to either the SC group or the CSC group. After a median follow-up of 38.6 (interquartile range, 23.5–62.1) months, PFS was significantly longer in the CSC (HR for PFS adjusted for stratification factors, 0.70; 95% CI: 0.52–0.95; stratified log-rank *p* = 0.023). The 3-year PFS rates were 66.3% (95% CI: 59.6–72.1) with CSC and 60.2% (95% CI: 53.6–66.3) with SC. Two Grade 5 adverse events (febrile neutropenia and dyspnea) occurred during neoadjuvant chemotherapy. Other than that, treatments were well tolerated.

This study showed the effectiveness of neoadjuvant DOS in Korea.

#### 2.2.3. RESOLVE

This study was conducted to evaluate the efficacy of perioperative (preoperative and postoperative) SOX (S-1 + oxaliplatin) compared with adjuvant (postoperative) CapeOX (Capecitabine + oxaliplatin) in patients with resectable GC undergoing gastrectomy with D2 lymph node dissection in China [30].

This study enrolled patients with histologically confirmed cT4a, N+, M0 or cT4b, N any, M0 gastric or gastroesophageal junction adenocarcinoma who underwent D2 gastrectomy. Patients were randomly assigned (1:1:1) to receive postoperative CapeOX (eight cycles), postoperative SOX (eight cycles), or perioperative SOX (three preoperative cycles and five postoperative cycles followed by three cycles of S-1 monotherapy). The primary endpoint was 3-year DFS to assess the potential superiority of perioperative SOX compared with postoperative SOX and the non-inferiority of postoperative SOX compared with postoperative CapeOX.

A total of 1022 patients were enrolled and randomly assigned to either the postoperative CapeOX group, the postoperative SOX group, or the perioperative SOX group. Three-year DFS was 51.1% (95% CI: 45.5–56.3) in the postoperative CapeOX group, 56.5% (95% CI: 51.0–61.7) in the postoperative SOX group, and 59.4% (95% CI: 53.8–64.6) in the perioperative SOX group. The HR was 0.77 (95% CI: 0.61–0.97; Wald *p* = 0.028) for the perioperative SOX group compared with the postoperative CapeOX group and 0.86 (95% CI: 0.68–1.07; Wald *p* = 0.17) for the postoperative SOX group compared with the postoperative CapeOX group. 

Perioperative SOX showed clinically meaningful benefits compared with postoperative CapeOX, and postoperative SOX was non-inferior to postoperative CapeOX.

**Table 3:** Pivotal randomized controlled clinical trial for neoadjuvant chemotherapy in East Asia.

Study	N	Published Year	Area	Setting	Disease Type	Recommended Resection	Treatment	Survival	HR	Reference
JCOG 0501	149	2021	Japan	Borrmann type 3 (≧8 cm)	gastric 100%	D2/3	NAC SP → Adj S-1	3-year OS: 62%	0.916	[27]
	151			Borrmann type 4	AC 100%		Adj S-1	3-year OS: 61%		
PRODIGY	238	2021	Korea	Stage II/III	gastric 94%,GEJ 6%	D2	NAC DOS → Adj S-1	3-year PFS: 66%	0.69	[29]
	246				AC 100%		Adj S-1	3-year PFS: 60%		
RESOLVE	337	2021	China	Stage III	gastric 64%, GEJ 36%	D2	Perioperative SOX	3-year DFS:59%	0.77	[30]
	340						Adj SOX	3-year DFS: 56%		
					AC 100%				0.86	
	345						Adj Capox	3-year DFS:51%	⁻	

GEJ—gastroesophageal junction; AC—adenocarcinoma; OS—overall survival; PFS—progression-free survival; DFS—disease-free survival; Capox—capecitabine and oxaliplatin; SOX—S-1 and oxaliplatin; HR—hazard ratio.

**Table 4:** Neoadjuvant chemotherapy regimens in East Asia.

Study	Regimen	Frequency	Drug	Dose		Period
**JCOG0501**	**SP**	4 weeks	S-1	40–80 mg/body, twice daily	days 1–21	Preoperative; 2 cycles
			Cisplatine	60 mg/m^2^	day 8	
	**S-1**	6 weeks	S-1	40–80 mg/body, twice daily	days 1–28	Postoperative; every 6 weeks for 1 year
**PRODIGY**	**DOS**	3 weeks	Docetaxel	50 mg/m^2^	day 1	Preoperative; 3 cycles
			Oxaliplatine	100 mg/m^2^	day 1	
			S-1	40–80 mg/body, twice daily	days 1–14	
	**S-1**	6 weeks	S-1	40–80 mg/body, twice daily	days 1–28	Postoperative; every 6 weeks for 1 year
**RESOLVE**	**SOX**	3 weeks	Oxaliplatine	130 mg/m^2^	day 1	Preoperative; 3 cycles
			S-1	40–80 mg/body, twice daily	days 1–14	Postoperative; 5 cycles
	**S-1**	3 weeks	S-1	40–80 mg/body, twice daily	days 1–14	After above; 3 cycles

#### 2.2.4. Final Notes

As mentioned above, perioperative chemotherapy is also being evaluated in East Asia.

In China, the RESONANCE trial, a phase III study, using SOX as NAC for clinical stages (cStage) IIA-IIIC, reported that 67.8% of patients receiving NAC showed pathological efficacy, and 23.6% showed complete response without significant differences in short-term surgical outcomes [31].

Treatment strategies may further change depending on the results of other ongoing studies.

## 3. Europe

### 3.1. Perioperative Chemotherapy in Europe (Table 5 and Table 6)

The European Society for Medical Oncology (ESMO) clinical practice guidelines suggest gastrectomy with D2 lymph node dissection be performed and recommend perioperative chemotherapy preferentially for patients with stage IB-III GC. 

This section summarizes pivotal clinical trials on perioperative chemotherapy in Europe.

#### 3.1.1. MAGIC

A regimen of epirubicin, cisplatin, and infused fluorouracil (ECF) has been reported to improve survival in patients with incurable locally advanced or metastatic GC in Europe [32,33,34]. However, numerous phase III trials at these times did not show the survival benefits of adjuvant chemotherapy.

The Medical Research Council Adjuvant Gastric Infusional Chemotherapy (MAGIC) trial was conducted to prove the efficacy of perioperative ECF for patients with potentially resectable GC [35]. 

This study enrolled patients with resectable adenocarcinoma of the stomach, esophagogastric junction, or lower esophagus. A total of 503 patients were randomly assigned to either the perioperative ECF group or the surgery-only group. The ECF regimen consisted of a 3-week cycle of intravenous epirubicin and cisplatin on day 1, plus continuous intravenous infusion of fluorouracil on days 1 to 21. Patients assigned to the ECF group received three preoperative and three postoperative cycles of ECF. The primary endpoint was OS.

This study showed a significant improvement in OS in the perioperative ECF group compared to the surgery-only group (HR for death, 0.75; 95% CI: 0.60-0.93; *p* = 0.009; 5-year survival rate, 36% vs. 23%), as well as an improvement in PFS (HR for progression, 0.66; 95% CI: 0.53–0.81; *p* < 0.001). The resected tumors were smaller and less advanced in the perioperative ECF group. The adverse effects of ECF were similar to those previously reported among patients with locally advanced and metastatic GC.

Based on the results of this study, ECF has become the standard of perioperative chemotherapy in Europe.

#### 3.1.2. FLOT4

ECF regimens have been considered standard perioperative chemotherapy. However, the overall survival rate is not satisfactory.

Docetaxel has shown efficacy in metastatic GC, both first-line (docetaxel, cisplatin, and fluorouracil (DCF)) [22] and second-line (docetaxel monotherapy) therapy [36]. However, the parental DCF regimen was associated with high toxicity, so a regimen was developed to replace cisplatin with oxaliplatin to reduce toxicities. The combination of fluorouracil, leucovorin, oxaliplatin, and docetaxel (FLOT) was evaluated for locally advanced and metastatic GCs and gastroesophageal junction adenocarcinoma in several phase II studies, and showed that FLOT was better tolerated than DCF and induced stronger tumor response than other regimens, including anthracycline-based triplets [37,38,39,40,41,42].

FLOT4 was conducted to evaluate the efficacy of FLOT as a perioperative chemotherapy for patients with locally advanced, resectable GC [43]. This study enrolled patients with histologically confirmed advanced clinical stage cT2-4 or nodal positive stage (cN+), or both, resectable tumors, with no evidence of distant metastases (cM0). Patients were randomly assigned to either the ECF/ECX group (three preoperative and three postoperative 3-week cycles of epirubicin and cisplatin on day 1 plus either fluorouracil or capecitabine on days 1 to 21) or the FLOT group (four preoperative and four postoperative 2-week cycles of docetaxel, oxaliplatin, leucovorin, and fluorouracil as a 24 h infusion on day 1). The primary endpoint was OS.

A total of 716 patients were randomly assigned to either the ECF/ECX group or the FLOT group. OS improved in the FLOT group compared with the ECF/ECX group (HR 0.77 (95% CI: 0.63–0.94); median OS, 50 months (38.33 to not reached) vs. 35 months (27.35 to 46.26)). The number of patients with related serious adverse effects was similar in the two groups (27% in the ECF/ECX group vs. 27% in the FLOT group). Hospitalization for toxicities was necessary in 26% of ECF/ECX patients and 25% of FLOT patients.

FLOT improved OS without increasing perioperative complications compared with perioperative ECF/ECX in resectable gastric or gastroesophageal junction adenocarcinoma.

**Table 5:** Pivotal randomized controlled clinical trial for perioperative therapy in Europe.

Study	N	Published Year	Area	Setting	Disease Type	Recommended Resection	Treatment	Survival	HR	Reference
MAGIC	250	2006	UK	No evidence of distant metastases, or locally advanced inoperable disease	gastric 74%, Lower esophageal/GEJ 26%	undefined	surgery	5-year OS: 23%	0.75	[35]
	253				AC 100%		ECF	5-year OS: 36%		
FLOT 4	360	2011	France	cT2 or higher nodal positive stage (cN+), or both	gastric 44%, GEJ 56%	D2	ECF	median OS: 35 months	0.77	[43]
	356				AC 100%		FLOT	median OS: 50 months		

GEJ—gastroesophageal junction; AC—adenocarcinoma; ECF—epirubicin, cisplatin and 5-fluorouracil; FLOT—fluorouracil, leucovorin, oxaliplatin and docetaxel; OS—overall survival; HR—hazard ratio.

**Table 6:** Perioperative chemotherapy regimens in Europe.

Regimen	Frequency	Drug	Dose		Period
**ECF**	3 weeks	Epirubicin	50 mg/m^2^	day 1	Preoperative; 3 cycles
		Cisplatine	60 mg/m^2^		
		Fluorouracil	200 mg/m^2^/day	days 1–21	Postoperative; 3 cycles
**ECX**	3 weeks	Epirubicin	50 mg/m^2^	day 1	Preoperative; 3 cycles
		Cisplatine	60 mg/m^2^		
		Capecitabine	1250 mg/m^2^	days 1–21	Postoperative; 3 cycles
**FLOT**	2 weeks	Docetaxel	50 mg/m^2^	day 1	Preoperative; 4 cycles
		Oxaliplatine	85 mg/m^2^		
		Leucovorin	200 mg/m^2^		Postoperative; 4 cycles
		Fluorouracil	2600 mg/m^2^, 24-h infusion		

#### 3.1.3. Final Notes

Perioperative chemotherapy using triplet regimens (fluorinated pyrimidines, platinum, and taxanes) is the standard treatment in Europe. However, ESMO guidelines recommend postoperative chemotherapy or chemoradiotherapy for patients who undergo gastrectomy without preoperative therapy [33]. Postoperative chemotherapy has been adopted in Europe based on the results of the ACTS-GC and CLASSIC studies. Although chemoradiotherapy has been established in North America, the ESMO guidelines indicate it is only for suboptimal surgery with less lymphadenectomy and suspicious micrometastases [44,45].

## 4. North America

### 4.1. Chemoradiotherapy in North America (Table 7 and Table 8)

Postoperative chemotherapy in East Asia and preoperative chemotherapy in Europe have been considered the standard treatments, but postoperative chemoradiation therapy has been considered the standard in North America. 

The National Comprehensive Cancer Network (NCCN) guidelines agree that gastrectomy with D2 lymph node dissection is associated with low mortality and reasonable survival benefits. However, the NCCN guidelines recommend gastrectomy with D1 or modified D2 lymph node dissection and also emphasize that D2 lymph node dissection should be performed only by experienced surgeons because of its technical difficulty [46,47].

Therefore, different perioperative treatments are recommended in North America compared to other regions. This section summarizes the situation with a focus on postoperative chemoradiotherapy.

**Table 7:** Pivotal randomized controlled clinical trial for postoperative chemoradiotherapy in North America.

Study	N	Published Year	Area	Setting	Disease Type	Recommended Resection	Treatment	Survival	HR	Reference
INT-0116	275	2001	US	Stage IB–IV M0	gastric 80%, GEJ 20%	D2 (D0 54%, D1 36%, D2 10%)	surgery	median OS: 27 months	1.35	[24]
	281				AC 100%		Fluorouracil + leucovorin + Radiation (45 Gy)	median OS: 36 months		

GEJ—gastroesophageal junction; AC—adenocarcinoma; OS—overall survival; HR—hazard ratio.

**Table 8:** Chemoradiotherapy regimen in North America.

Regimen	Frequency	Drug	Dose		Period
**INT-0116**	4 weeks	5-FU	425 mg/m^2^	days 1–5	Preradiotherapy; 1 cycle
		Leucovorin	20 mg/m^2^		
	5 weeks	Radiotherapy	45 Gy/25 Fr	5 days a week, over 5 weeks	
		5-FU	400 mg/m^2^	the first 4 days and the last 3 days of radiotherapy	
		Leucovorin	20 mg/m^2^		
	4 weeks	5-FU	425 mg/m^2^	days 1–5	Postradiotherapy; 2 cycles
		Leucovorin	20 mg/m^2^		

#### 4.1.1. SWOG9008/INT-0116

The curative treatment for GC is resection, but most patients were not successfully treated via surgery alone in the 1990s in North America. This study was designed to prove the efficacy of postoperative chemoradiotherapy for patients with resectable adenocarcinoma [24]. 

A total of 556 patients with resected adenocarcinoma of the stomach or gastroesophageal junction were randomized to the surgery plus postoperative chemoradiotherapy group or the surgery-only group. The adjuvant treatment consisted of fluorouracil plus 20 mg/m^2^/day of leucovorin, for five days, followed by 4500 cGy of radiation at 180 cGy/day, administered five days per week for five weeks, with modified doses of fluorouracil and leucovorin on the first four and last three days of radiotherapy. 

One month after the completion of radiotherapy, two five-day cycles of fluorouracil plus leucovorin were administered one month apart.

The median OS in the surgery-only group was 27 months compared with 36 months in the chemoradiotherapy group (death HR was 1.35 (95% CI: 1.09–1.66; *p* = 0.005)). The HR for relapse was 1.52 (95% CI: 1.23–1.86; *p* < 0.001). Three patients (1%) died due to chemoradiotherapy toxicities; grade 3 toxicities occurred in 41% of the patients in the chemoradiotherapy group, and grade 4 toxicities occurred in 32% of patients.

In a later updated analysis, this study demonstrated strong persistent benefits from postoperative chemoradiotherapy [48]. This study established postoperative chemoradiotherapy in North America. The NCCN guidelines now recommend it only for patients who undergo gastrectomy with less than a D2 lymph node dissection [49]. 

#### 4.1.2. Final Notes

Postoperative chemoradiotherapy was established in North America and is still recommended in certain cases. Perioperative chemotherapy is also recommended, according to the results of the FLOT4 study [43]. Regarding postoperative chemotherapy, the NCCN guidelines state that it is difficult to apply the results of the ACTS-GC and CLASSIC studies because gastrectomy with D2 lymph node dissection is rarely performed in North American hospitals [50]. Postoperative chemotherapy with XELOX is included only as an option for patients who undergo D2 or modified D2 lymph node dissection [51]. In North America, perioperative treatment is selected based on the circumstances described above.

## 5. New Agents for Adjuvant Chemotherapy

In recent years, new drugs such as molecular-targeted drugs and ICIs have improved the prognosis of GC [52,53,54,55]. Next, we summarize the trials of perioperative treatment using these agents. Many of these trials were designed around existing regimens containing fluoropyrimidines, platinum, and taxanes. Also, unlike previous trials, these were conducted across multiple regions.

### 5.1. Molecular-Targeted Drugs (Table 9)

The RAMSES-AIO study examined the utility of a trial treatment adding ramucirumab to perioperative FLOT therapy [56]. The results suggested that the addition of ramucirumab may increase the R0 resection rate.

The PETRARCA-AIO study examined the addition of trastuzumab to conventional FLOT for HER2-positive cancer, and the results suggested an increase in pCR and nodal negativity rates [57]. 

EORTC also conducted the INNOVATION study to verify the efficacy of perioperative chemotherapy, comparing XP/FP only to experimental treatments with the addition of trastuzumab or trastuzumab and pertuzumab for HER2-positive GC [58].

**Table 9:** New agents for adjuvant chemotherapy/Molecular-targeted drugs.

Study	Regimen	Frequency	Drug	Dose		Period
**RAMSES**	**FLOT**	2 weeks	Docetaxel	50 mg/m^2^	day 1	Preoperative; 4 cycles
			Oxaliplatine	85 mg/m^2^		
			Leucovorin	200 mg/m^2^		
			Fluorouracil	2600 mg/m^2^, 24 h infusion		Postoperative; 4 cycles
	**Ramcirumab**		8 mg/kg		day 1	
	**Ramcirumab**	2 weeks	8 mg/kg		day 1	After above; 16 cycles
**PETRARCA**	**FLOT**	2 weeks	Docetaxel	50 mg/m^2^	day 1	Preoperative; 4 cycles
			Oxaliplatine	85 mg/m^2^		
			Leucovorin	200 mg/m^2^		Postoperative; 4 cycles
			Fluorouracil	2600 mg/m^2^, 24 h infusion		
	**Trastuzumab**	3 weeks	[Initial dose] 8 mg/kg		day 1	Preoperative; 3 cycles
			[Maintenance dose] 6 mg/kg			Postoperative; 3 cycles
	**Perutuzumab**		840 mg			After above; 9 cycles
**INNOVATION**	**XELOX (CapOx)**	3 weeks	Capecitabine	1000 mg/m^2^, twice daily	days 1–14	Preoperative; 3 cycles
			Oxaliplatine	130 mg/m^2^	day 1	
	**XP**		Capecitabine	1000 mg/m^2^, twice daily	days 1–14	
			Cisplatine	80 mg/m^2^	day 1	Postoperative; 3 cycles
	**FP**		Fluorouracil	800 mg/m^2^	days 1–5	
			Cisplatine	80 mg/m^2^	day 1	
	**FLOT**	2 weeks	Docetaxel	50 mg/m^2^	day 1	Preoperative; 4 cycles
			Oxaliplatine	85 mg/m^2^		
			Leucovorin	200 mg/m^2^		
			Fluorouracil	2600 mg/m^2^, 24 h infusion		
	**FOLFOX**		Oxaliplatine	85 mg/m^2^	day 1	Postoperative; 4 cycles
			Leucovorin	400 mg/m^2^		
			Fluorouracil	[bolus] 400 mg/m^2^		
				[continuous infusion] 2400 mg/m^2^, 46–48 h		
	**Any of the above regimens combined with any of the following regimens**					
	**Trastuzumab**	3 weeks	[Initial dose] 8 mg/kg		day 1	
			[Maintenance dose] 6 mg/kg			Preoperative; 3 cycles
	**Perutuzumab**		840 mg			Postoperative; 3 cycles
	**Trastuzumab**		[Initial dose] 8 mg/kg		day 1	After above; 9 cycles
			[Maintenance dose] 6 mg/kg			

### 5.2. Immune Checkpoint Inhibitors (Table 10)

The usefulness of immune checkpoint inhibitors has been tested in perioperative treatment as well as in palliative chemotherapy.

The KEYNOTE 585 study evaluated the addition of pembrolizumab to perioperative XP/FP/FLOT [59], and the MATTERHORN study evaluated the addition of durvalumab to perioperative FLOT [60]. 

In addition, the DANTE study examined the benefits of adding atezolizumab to perioperative FLOT [61]. 

The results of these clinical trials are expected to change future treatment strategies for GC.

**Table 10:** New agents for adjuvant chemotherapy/Immune checkpoint inhibitors.

Study	Regimen	Frequency	Drug	Dose		Period
**KEYNOTE-585**	**FLOT**	2 weeks	Docetaxel	50 mg/m^2^	day 1	Preoperative; 4 cycles
			Oxaliplatine	85 mg/m^2^		
			Leucovorin	200 mg/m^2^		Postoperative; 4 cycles
			Fluorouracil	2600 mg/m^2^, 24 h infusion		
	**XP**	3 weeks	Capecitabine	1000 mg/m^2^, twice daily	day 1–14	Preoperative; 3 cycles
			Cisplatine	80 mg/m^2^	day 1	
	**FP**		Fluorouracil	800 mg/m^2^	day 1–5	Postoperative; 3 cycles
			Cisplatine	80 mg/m^2^	day 1	
	**Any of the above regimens combined with the following regimen**					
	**Pembrolizumab**	3 weeks	200 mg		day 1	Preoperative; 3 cycles
						Postoperative; 3 cycles
						After above; 11 additional cycles
**MATTERHORN**	**FLOT**	4 weeks	Docetaxel	50 mg/m^2^	day 1, 15	Preoperative; 2 cycles
			Oxaliplatine	85 mg/m^2^		
			Leucovorin	200 mg/m^2^		
			Fluorouracil	2600 mg/m^2^, 24 h infusion		Postoperative; 2 cycles
	**Durvalumab**		1500 mg		day 1	
	**Durvalumab**	4 weeks	1500 mg		day 1	After above; 10 additional cycles
**DANTE**	**FLOT**	2 weeks	Docetaxel	50 mg/m^2^	day 1	Preoperative; 4 cycles
			Oxaliplatine	85 mg/m^2^		
			Leucovorin	200 mg/m^2^		
			Fluorouracil	2600 mg/m^2^, 24 h infusion		
	**FLOT**	2 weeks	Docetaxel	50 mg/m^2^	day 1	Postoperative; 4 cycles
			Oxaliplatine	85 mg/m^2^		
			Leucovorin	200 mg/m^2^		
			Fluorouracil	2600 mg/m^2^, 24 h infusion		
	**Atezolizumab**		840 mg		day 1	
	**Atezolizumab**	3 weeks	1200 mg		day 1	After above; 8 additional cycles

## 6. Conclusions

The perioperative treatment of GC has undergone significant development in various regions since the 2000s. Perioperative regimens were initially established by drawing from the treatment protocols used for metastatic GC. Consequently, treatment advancements began with S-1 in East Asia and ECF in Europe. Variations in surgical techniques may have also influenced the intensity of chemotherapy, including whether doublet or triplet regimens were employed, as well as the incorporation of radiation therapy. Particularly, in East Asia, more trials have been conducted than in other regions due to the large number of GCs. In this way, perioperative chemotherapy has evolved independently in each region. Now, perioperative chemotherapy based on fluorinated pyrimidines with added platinum and taxanes has been established in all regions with improved outcomes. Perioperative therapy using novel agents has also been tested and evaluated in the three major regions with promising results. Perioperative chemotherapy has become an integral part of many treatment strategies and requires continued research and evaluation.

## Data Availability

No new data were created or analyzed in this study. Data sharing is not applicable to this article.

## References

[1] Sung H., Ferlay J., Siegel R.L., Laversanne M., Soerjomataram I., Jemal A., Bray F. (2021). Global Cancer Statistics 2020: GLOBOCAN Estimates of Incidence and Mortality Worldwide for 36 Cancers in 185 Countries. CA Cancer J. Clin..

[2] Lin J.-X., Tang Y.-H., Lin G.-J., Ma Y.-B., Desiderio J., Li P., Xie J.-W., Wang J.-B., Lu J., Chen Q.-Y. (2022). Association of Adjuvant Chemotherapy With Overall Survival Among Patients With Locally Advanced Gastric Cancer After Neoadjuvant Chemotherapy. JAMA Netw. Open.

[3] Tegels J.J., De Maat M.F., Hulsewé K.W., Hoofwijk A.G., Stoot J.H. (2014). Improving the outcomes in gastric cancer surgery. World J. Gastroenterol..

[4] Magbanua M., Swigart L., Wu H.-T., Hirst G., Yau C., Wolf D., Tin A., Salari R., Shchegrova S., Pawar H. (2021). Circulating tumor DNA in neoadjuvant-treated breast cancer reflects response and survival. Ann. Oncol..

[5] Powles T., Assaf Z.J., Davarpanah N., Banchereau R., Szabados B.E., Yuen K.C., Grivas P., Hussain M., Oudard S., Gschwend J.E. (2021). ctDNA guiding adjuvant immunotherapy in urothelial carcinoma. Nature.

[6] Loupakis F., Sharma S., Derouazi M., Murgioni S., Biason P., Rizzato M.D., Rasola C., Renner D., Shchegrova S., Malashevich A.K. (2021). Detection of Molecular Residual Disease Using Personalized Circulating Tumor DNA Assay in Patients With Colorectal Cancer Undergoing Resection of Metastases. JCO Precis. Oncol..

[7] Reinert T., Henriksen T.V., Christensen E., Sharma S., Salari R., Sethi H., Knudsen M., Nordentoft I.K., Wu H.-T., Tin A.S. (2019). Analysis of Plasma Cell-Free DNA by Ultradeep Sequencing in Patients With Stages I to III Colorectal Cancer. JAMA Oncol..

[8] Mencel J., Slater S., Cartwright E., Starling N. (2022). The Role of ctDNA in Gastric Cancer. Cancers.

[9] Huffman B.M., Aushev V.N., Budde G.L., Chao J., Dayyani F., Hanna D., Botta G.P., Catenacci D.V., Maron S.B., Krinshpun S. (2022). Analysis of Circulating Tumor DNA to Predict Risk of Recurrence in Patients With Esophageal and Gastric Cancers. JCO Precis. Oncol..

[10] Kim Y.-W., Song Y., Kim H.-S., Sim H.W., Poojan S., Eom B.W., Kook M.-C., Joo J., Hong K.-M. (2019). Monitoring circulating tumor DNA by analyzing personalized cancer-specific rearrangements to detect recurrence in gastric cancer. Exp. Mol. Med..

[11] Leal A., van Grieken N.C.T., Palsgrove D.N., Phallen J., Medina J.E., Hruban C., Broeckaert M.A.M., Anagnostou V., Adleff V., Bruhm D.C. (2020). White blood cell and cell-free DNA analyses for detection of residual disease in gastric cancer. Nat. Commun..

[12] Sakuramoto S., Sasako M., Yamaguchi T., Kinoshita T., Fujii M., Nashimoto A., Furukawa H., Nakajima T., Ohashi Y., Imamura H. (2007). Adjuvant Chemotherapy for Gastric Cancer with S-1, an Oral Fluoropyrimidine. New Engl. J. Med..

[13] Sasako M., Sakuramoto S., Katai H., Kinoshita T., Furukawa H., Yamaguchi T., Nashimoto A., Fujii M., Nakajima T., Ohashi Y. (2011). Five-Year Outcomes of a Randomized Phase III Trial Comparing Adjuvant Chemotherapy With S-1 Versus Surgery Alone in Stage II or III Gastric Cancer. J. Clin. Oncol..

[14] Tsuji Y. (2018). Adjuvant Chemotherapy: Current Practices, Efficacy and Future Directions. Cancer Etiology, Diagnosis and Treatments.

[15] Bang Y.-J., Kim Y.-W., Yang H.-K., Chung H.C., Park Y.-K., Lee K.H., Lee K.-W., Kim Y.H., Noh S.-I., Cho J.Y. (2012). Adjuvant capecitabine and oxaliplatin for gastric cancer after D2 gastrectomy (CLASSIC): A phase 3 open-label, randomised controlled trial. Lancet.

[16] Noh S.H., Park S.R., Yang H.-K., Chung H.C., Chung I.-J., Kim S.-W., Kim H.-H., Choi J.-H., Kim H.-K., Yu W. (2014). Adjuvant capecitabine plus oxaliplatin for gastric cancer after D2 gastrectomy (CLASSIC): 5-year follow-up of an open-label, randomised phase 3 trial. Lancet Oncol..

[17] Taguchi T., Sakata Y., Kanamaru R., Kurihara M., Suminaga M., Ota J., Hirabayashi N. (1998). Late phase II clinical study of RP56976 (docetaxel) in patients with advanced/recurrent gastric cancer: A Japanese Cooperative Study Group trial (group A). Gan To Kagaku Ryoho.

[18] Yoshida K., Hirabayashi N., Takiyama W., Ninomiya M., Takakura N., Sakamoto J., Nishiyama M., Toge T. (2004). Phase I study of combination therapy with S-1 and docetaxel (TXT) for advanced or recurrent gastric cancer. Anticancer Res..

[19] Yoshida K., Ninomiya M., Takakura N., Hirabayashi N., Takiyama W., Sato Y., Todo S., Terashima M., Gotoh M., Sakamoto J. (2006). Phase II Study of Docetaxel and S-1 Combination Therapy for Advanced or Recurrent Gastric Cancer. Clin. Cancer Res..

[20] Koizumi W., Kim Y.H., Fujii M., Kim H.K., Imamura H., Lee K.H., Hara T., Chung H.C., Satoh T., The JACCRO and KCSG Study Group (2014). Addition of docetaxel to S-1 without platinum prolongs survival of patients with advanced gastric cancer: A randomized study (START). J. Cancer Res. Clin. Oncol..

[21] Wada Y., Yoshida K., Suzuki T., Mizuiri H., Konishi K., Ukon K., Tanabe K., Sakata Y., Fukushima M. (2006). Synergistic effects of docetaxel and S-1 by modulating the expression of metabolic enzymes of 5-fluorouracil in human gastric cancer cell lines. Int. J. Cancer.

[22] Van Cutsem E., Moiseyenko V.M., Tjulandin S., Majlis A., Constenla M., Boni C., Rodrigues A., Fodor M., Chao Y., Voznyi E. (2006). Phase III Study of Docetaxel and Cisplatin Plus Fluorouracil Compared With Cisplatin and Fluorouracil As First-Line Therapy for Advanced Gastric Cancer: A Report of the V325 Study Group. J. Clin. Oncol..

[23] Yoshida K., Kodera Y., Kochi M., Ichikawa W., Kakeji Y., Sano T., Nagao N., Takahashi M., Takagane A., Watanabe T. (2019). Addition of Docetaxel to Oral Fluoropyrimidine Improves Efficacy in Patients With Stage III Gastric Cancer: Interim Analysis of JACCRO GC-07, a Randomized Controlled Trial. J. Clin. Oncol..

[24] Macdonald J.S., Smalley S.R., Benedetti J., Hundahl S.A., Estes N.C., Stemmermann G.N., Haller D.G., Ajani J.A., Gunderson L.L., Jessup J.M. (2001). Chemoradiotherapy after Surgery Compared with Surgery Alone for Adenocarcinoma of the Stomach or Gastroesophageal Junction. N. Engl. J. Med..

[25] Kim S., Lim D.H., Lee J., Kang W.K., MacDonald J.S., Park C.H., Park S.H., Lee S.-H., Kim K., Park J.O. (2005). An observational study suggesting clinical benefit for adjuvant postoperative chemoradiation in a population of over 500 cases after gastric resection with D2 nodal dissection for adenocarcinoma of the stomach. Int. J. Radiat. Oncol..

[26] Park S.H., Lim D.H., Sohn T.S., Lee J., Zang D.Y., Kim S.T., Kang J.H., Oh S.Y., Hwang I.G., Ji J.H. (2021). A randomized phase III trial comparing adjuvant single-agent S1, S-1 with oxaliplatin, and postoperative chemoradiation with S-1 and oxaliplatin in patients with node-positive gastric cancer after D2 resection: The ARTIST 2 trial. Ann. Oncol..

[27] Iwasaki Y., Terashima M., Mizusawa J., Katayama H., Nakamura K., Katai H., Yoshikawa T., Ito S., Kaji M., Kimura Y. (2021). Gastrectomy with or without neoadjuvant S-1 plus cisplatin for type 4 or large type 3 gastric cancer (JCOG0501): An open-label, phase 3, randomized controlled trial. Gastric Cancer.

[28] Association J.G.C. (2021). Japanese gastric cancer treatment guidelines 2021 (6th edition). Gastric Cancer.

[29] Kang Y.-K., Yook J.H., Park Y.-K., Lee J.S., Kim Y.-W., Kim J.Y., Ryu M.-H., Rha S.Y., Chung I.J., Kim I.-H. (2021). PRODIGY: A Phase III Study of Neoadjuvant Docetaxel, Oxaliplatin, and S-1 Plus Surgery and Adjuvant S-1 Versus Surgery and Adjuvant S-1 for Resectable Advanced Gastric Cancer. J. Clin. Oncol..

[30] Zhang X., Liang H., Li Z., Xue Y., Wang Y., Zhou Z., Yu J., Bu Z., Chen L., Du Y. (2021). Perioperative or postoperative adjuvant oxaliplatin with S-1 versus adjuvant oxaliplatin with capecitabine in patients with locally advanced gastric or gastro-oesophageal junction adenocarcinoma undergoing D2 gastrectomy (RESOLVE): An open-label, superiority and non-inferiority, phase 3 randomised controlled trial. Lancet Oncol..

[31] Wang X., Li S., Xie T., Lu Y., Guo X., Lin C. (2020). Early results of the randomized, multicenter, controlled evaluation of S-1 and oxaliplatin as neoadjuvant chemotherapy for Chinese advanced gastric cancer patients (RESONANCE Trial). J. Clin. Oncol..

[32] Findlay M., Cunningham D., Norman A., Mansi J., Nicolson M., Hickish T., Nicolson V., Nash A., Sacks N., Ford H. (1994). A phase II study in advanced gastro-esophageal cancer using epirubicin and cisplatin in combination with continuous infusion 5-fluorouracil (ECF). Ann. Oncol..

[33] Webb A., Cunningham D., Scarffe J.H., Harper P., Norman A., Joffe J.K., Hughes M., Mansi J., Findlay M., Hill A. (1997). Randomized trial comparing epirubicin, cisplatin, and fluorouracil versus fluorouracil, doxorubicin, and methotrexate in advanced esophagogastric cancer. J. Clin. Oncol..

[34] Ross P., Nicolson M., Cunningham D., Valle J., Seymour M., Harper P., Price T., Anderson H., Iveson T., Hickish T. (2002). Prospective Randomized Trial Comparing Mitomycin, Cisplatin, and Protracted Venous-Infusion Fluorouracil (PVI 5-FU) With Epirubicin, Cisplatin, and PVI 5-FU in Advanced Esophagogastric Cancer. J. Clin. Oncol..

[35] Cunningham D., Allum W.H., Stenning S.P., Thompson J.N., Van de Velde C.J., Nicolson M., Scarffe J.H., Lofts F.J., Falk S.J., Iveson T.J. (2006). Perioperative Chemotherapy versus Surgery Alone for Resectable Gastroesophageal Cancer. New Engl. J. Med..

[36] Ford H.E.R., Marshall A., Bridgewater A.J., Janowitz T., Coxon F.Y., Wadsley J., Mansoor W., Fyfe D., Madhusudan S., Middleton G.W. (2014). Docetaxel versus active symptom control for refractory oesophagogastric adenocarcinoma (COUGAR-02): An open-label, phase 3 randomised controlled trial. Lancet Oncol..

[37] Al-Batran S.-E., Hartmann J.T., Hofheinz R., Homann N., Rethwisch V., Probst S., Stoehlmacher J., Clemens M.R., Mahlberg R., Fritz M. (2008). Biweekly fluorouracil, leucovorin, oxaliplatin, and docetaxel (FLOT) for patients with metastatic adenocarcinoma of the stomach or esophagogastric junction: A phase II trial of the Arbeitsgemeinschaft Internistische Onkologie. Ann. Oncol..

[38] Al-Batran S.-E., Pauligk C., Homann N., Hartmann J.T., Moehler M., Probst S., Rethwisch V., Stoehlmacher-Williams J., Prasnikar N., Hollerbach S. (2013). The feasibility of triple-drug chemotherapy combination in older adult patients with oesophagogastric cancer: A randomised trial of the Arbeitsgemeinschaft Internistische Onkologie (FLOT65+). Eur. J. Cancer.

[39] Al-Batran S.E., Homann N., Pauligk C., Illerhaus G., Martens U.M., Stoehlmacher J., Schmalenberg H., Luley K.B., Prasnikar N., Egger M. (2017). Effect of Neoadjuvant Chemotherapy Followed by Surgical Resection on Survival in Patients With Limited Metastatic Gastric or Gastroesophageal Junction Cancer: The AIO-FLOT3 Trial. JAMA Oncol..

[40] Homann N., Pauligk C., Luley K., Kraus T.W., Bruch H.-P., Atmaca A., Noack F., Altmannsberger H.-M., Jäger E., Al-Batran S.-E. (2012). Pathological complete remission in patients with oesophagogastric cancer receiving preoperative 5-fluorouracil, oxaliplatin and docetaxel. Int. J. Cancer.

[41] Schulz C., Kullmann F., Kunzmann V., Fuchs M., Geissler M., Vehling-Kaiser U., Stauder H., Wein A., Al-Batran S.-E., Kubin T. (2015). NeoFLOT: Multicenter phase II study of perioperative chemotherapy in resectable adenocarcinoma of the gastroesophageal junction or gastric adenocarcinoma-Very good response predominantly in patients with intestinal type tumors. Int. J. Cancer.

[42] Becker K., Mueller J.D., Schulmacher C., Ott K., Fink U., Busch R., Bottcher K., Siewert J.R., Hofler H. (2003). Histomorphology and grading of regression in gastric carcinoma treated with neoadjuvant chemotherapy. Cancer.

[43] Al-Batran S.-E., Homann N., Pauligk C., Goetze T.O., Meiler J., Kasper S., Kopp H.-G., Mayer F., Haag G.M., Luley K. (2019). Perioperative chemotherapy with fluorouracil plus leucovorin, oxaliplatin, and docetaxel versus fluorouracil or capecitabine plus cisplatin and epirubicin for locally advanced, resectable gastric or gastro-oesophageal junction adenocarcinoma (FLOT4): A randomised, phase 2/3 trial. Lancet.

[44] Kim I.-H. (2019). Current status of adjuvant chemotherapy for gastric cancer. World J. Gastrointest. Oncol..

[45] Dikken J.L., Jansen E.P., Cats A., Bakker B., Hartgrink H.H., Kranenbarg E.M.-K., Boot H., Putter H., Peeters K.C., van de Velde C.J. (2010). Impact of the Extent of Surgery and Postoperative Chemoradiotherapy on Recurrence Patterns in Gastric Cancer. J. Clin. Oncol..

[46] Stiekema J., Trip A.K., Jansen E.P.M., Boot H., Cats A., Ponz O.B., Verheij M., van Sandick J.W. (2014). The Prognostic Significance of an R1 Resection in Gastric Cancer Patients Treated with Adjuvant Chemoradiotherapy. Ann. Surg. Oncol..

[47] Schwarz R.E., Smith D.D. (2007). Clinical impact of lymphadenectomy extent in resectable gastric cancer of advanced stage. Ann. Surg. Oncol..

[48] Smalley S.R., Benedetti J.K., Haller D.G., Hundahl S.A., Estes N.C., Ajani J.A., Gunderson L.L., Goldman B., Martenson J.A., Jessup J.M. (2012). Updated analysis of SWOG-directed intergroup study 0116: A phase III trial of adjuvant radiochemotherapy versus observation after curative gastric cancer resection. J. Clin. Oncol..

[49] (2023). National Comprehensive Cancer Network (NCCN): NCCN Clinical Practice Guidelines in Oncology: Gastric Cancer Version 1. https://www.nccn.org/professionals/physician_gls/pdf/gastric.pdf.

[50] Songun I., Putter H., Kranenbarg E.M.-K., Sasako M., van de Velde C.J. (2010). Surgical treatment of gastric cancer: 15-year follow-up results of the randomised nationwide Dutch D1D2 trial. Lancet Oncol..

[51] Lee J., Lim D.H., Kim S., Park S.H., Park J.O., Park Y.S., Lim H.Y., Choi M.G., Sohn T.S., Noh J.H. (2012). Phase III Trial Comparing Capecitabine Plus Cisplatin Versus Capecitabine Plus Cisplatin With Concurrent Capecitabine Radiotherapy in Completely Resected Gastric Cancer With D2 Lymph Node Dissection: The ARTIST Trial. J. Clin. Oncol..

[52] Shitara K., Van Cutsem E., Bang Y.-J., Fuchs C., Wyrwicz L., Lee K.-W., Kudaba I., Garrido M., Chung H.C., Lee J. (2020). Efficacy and Safety of Pembrolizumab or Pembrolizumab Plus Chemotherapy vs Chemotherapy Alone for Patients With First-line, Advanced Gastric Cancer: The KEYNOTE-062 Phase 3 Randomized Clinical Trial. JAMA Oncol..

[53] Moehler M., Shitara K., Garrido M., Salman P., Shen L., Wyrwicz L., Yamaguchi K., Skoczylas T., Bragagnoli A.C., Liu T. (2020). LBA6_PR Nivolumab (nivo) plus chemotherapy (chemo) versus chemo as first-line (1L) treatment for advanced gastric cancer/gastroesophageal junction cancer (GC/GEJC)/esophageal adenocarcinoma (EAC): First results of the CheckMate 649 study. Ann. Oncol..

[54] Shitara K., Bang Y.-J., Iwasa S., Sugimoto N., Ryu M.-H., Sakai D., Chung H.-C., Kawakami H., Yabusaki H., Lee J. (2020). Trastuzumab Deruxtecan in Previously Treated HER2-Positive Gastric Cancer. N. Engl. J. Med..

[55] Boku N., Ryu M., Oh D.-Y., Oh S., Chung H., Lee K.-W., Omori T., Shitara K., Sakuramoto S., Chung I. (2020). LBA7_PR Nivolumab plus chemotherapy versus chemotherapy alone in patients with previously untreated advanced or recurrent gastric/gastroesophageal junction (G/GEJ) cancer: ATTRACTION-4 (ONO-4538-37) study. Ann. Oncol..

[56] Al-Batran S.-E., Hofheinz R., Schmalenberg H., Strumberg D., Goekkurt E., Angermeier S., Zander T., Potenberg J., Kopp H.-G., Pink D. (2020). 1424MO Perioperative FLOT plus ramucirumab versus FLOT alone for resectable esophagogastric adenocarcinoma—Updated results and subgroup analyses of the randomized phase II/III trial RAMSES/FLOT7 of the German AIO and Italian GOIM. Ann. Oncol..

[57] Al-Batran S.-E., Haag G., Ettrich T., Borchert K., Kretzschmar A., Teschendorf C., Siegler G., Ebert M., Goekkurt E., Welslau M. (2020). 1421MO Final results and subgroup analysis of the PETRARCA randomized phase II AIO trial: Perioperative trastuzumab and pertuzumab in combination with FLOT versus FLOT alone for HER2 positive resectable esophagogastric adenocarcinoma. Ann. Oncol..

[58] Wagner A.D., Grabsch H.I., Mauer M., Marreaud S., Caballero C., Thuss-Patience P., Mueller L., Elme A., Moehler M.H., Martens U. (2019). EORTC-1203-GITCG—The “INNOVATION”-trial: Effect of chemotherapy alone versus chemotherapy plus trastuzumab, versus chemotherapy plus trastuzumab plus pertuzumab, in the perioperative treatment of HER2 positive, gastric and gastroesophageal junction adenocarcinoma on pathologic response rate: A randomized phase II-intergroup trial of the EORTC-Gastrointestinal Tract Cancer Group, Korean Cancer Study Group and Dutch Upper GI-Cancer group. BMC Cancer.

[59] Bang Y.-J., Van Cutsem E., Fuchs C.S., Ohtsu A., Tabernero J., Ilson D.H., Hyung W.J., Strong E.V., Goetze T.O., Yoshikawa T. (2019). KEYNOTE-585: Phase III study of perioperative chemotherapy with or without pembrolizumab for gastric cancer. Futur. Oncol..

[60] Janjigian Y.Y., Van Cutsem E., Muro K., Wainberg Z., Al-Batran S.-E., Hyung W.J., Molena D., Marcovitz M., Ruscica D., Robbins S.H. (2022). MATTERHORN: Phase III study of durvalumab plus FLOT chemotherapy in resectable gastric/gastroesophageal junction cancer. Futur. Oncol..

[61] Homann N., Lorenzen S., Schenk M., Thuss-Patience P.C., Goekkurt E., Hofheinz R.D., Kretzschmar A., Bolling C., Angermeier S., Wicki A. (2020). Interim safety analysis of the DANTE trial: Perioperative atezolizumab in combination with FLOT versus FLOT alone in patients with resectable esophagogastric adenocarcinoma—A randomized, open-label phase II trial of the German Gastric Group at the AIO and SAKK. J. Clin. Oncol..

